# Hedgehog Signaling in Prostate Development, Regeneration and Cancer

**DOI:** 10.3390/jdb4040030

**Published:** 2016-10-19

**Authors:** Wade Bushman

**Affiliations:** Department of Urology, Carbone Comprehensive Cancer Center, University of Wisconsin, Madison, WI 53705, USA; bushman@urology.wisc.edu; Tel.: +1-608-262-5440; Fax: +1-608-262-6453

**Keywords:** Hedgehog, prostate, cancer

## Abstract

The prostate is a developmental model system study of prostate growth regulation. Historically the research focus was on androgen regulation of development and growth and instructive interactions between the mesenchyme and epithelium. The study of Hh signaling in prostate development revealed important roles in ductal morphogenesis and in epithelial growth regulation that appear to be recapitulated in prostate cancer. This overview of Hh signaling in the prostate will address the well-described role of paracrine signaling prostate development as well as new evidence suggesting a role for autocrine signaling, the role of Hh signaling in prostate regeneration and reiterative activities in prostate cancer.

## 1. Prostate Development

The prostate is a male sex-accessory gland that contributes secretions to the ejaculate. The mouse prostate is comprised of a highly branched ductal network in a loose fibro-vascular stroma arranged into anterior, dorsolateral and ventral lobes. The overall appearance and ratio of epithelium and stroma varies significantly between species but the basic mechanisms controlling prostate development appear to be conserved as evidenced by tissue recombination studies using mouse, rat, rabbit, and human prostate mesenchyme and epithelia [1]. The most widely used animal model for prostate development and morphogenesis is the mouse. 

The prostate develops from the urogenital sinus (UGS), a derivative of endodermal tissue. The first morphological event in prostate development is budding of urogenital sinus epithelium (UGE) into the surrounding urogenital sinus mesenchyme (UGM). This occurs in the mouse at embryonic day 16.5 (E16.5). During prenatal development, these epithelial buds elongate and form solid, unbranched ducts. Postnatal, the solid epithelial ducts elongate, canalize, and undergo branching morphogenesis. Extensive branching occurs by postnatal day 15 (P15) and is largely completed by P30 [2]. This morphogenetic process results in a mature prostate with an intricate branched ductal system that consists of three paired lobes: the ventral prostate (VP), the anterior prostate (AP), and the dorsolateral prostate (DLP). Each prostatic lobe has a distinct branching pattern [2].

## 2. Hedgehog Signaling in the Developing Prostate

**Figure 1:**
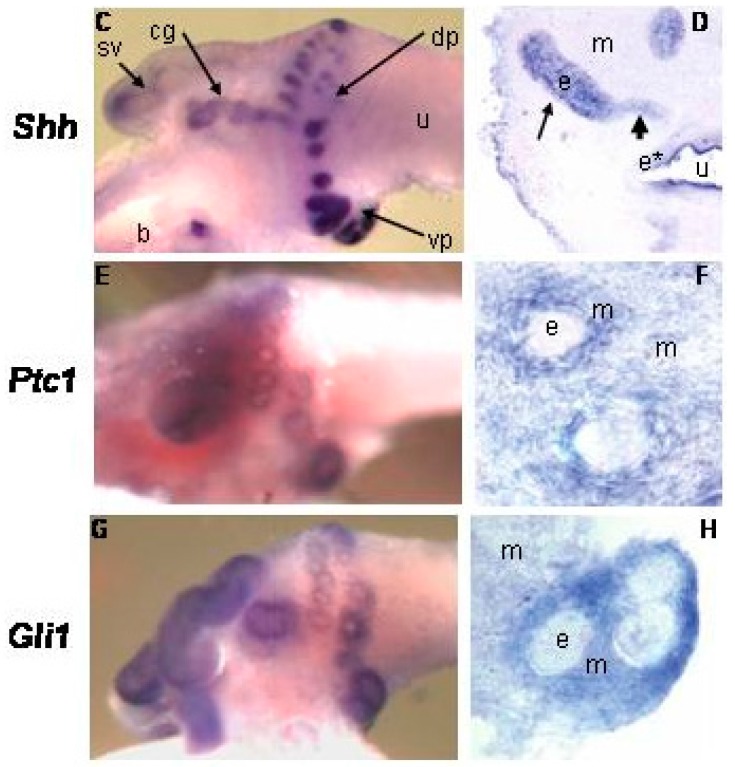
Whole mount in situ hybridization and sections of the P1 urogenital sinus (UGS). (m) mesenchyme (e) epithelium [4].

In the developing prostate, Shh and Ihh are expressed by the UGS epithelium and induce Hh target gene expression in the adjacent mesenchyme (Figure 1; [3,4,5,6,7]). Mesenchymal target genes include the conserved Hh targets Ptc, Gli1, Ptc2 and Hip1 [5] but also include a number of specific genes such as IGFBP-6, Angiopoetin4 (Agpt4), IGFBP-3, Inhibin beta-B and TIMP3 [8,9]. Several investigators noted minor expression of Ptc and Gli1 at the tips of the growing ducts suggesting a minor component of autocrine signaling [10,11]. 

## 3. Requirement for Prostate Development

*Shh* is the most abundantly expressed *Hh* ligand in the developing prostate [12]. *Shh* expression in the UGS epithelium increases with the onset of ductal budding. Abundant gene expression is maintained until the beginning of ductal branching at which point *Shh* begins to steadily decrease and eventually taper off to a low but detectable level in the adult [3,4]. *Shh* expression is localized to the epithelium at the distal tip as the ducts elongate and branch. During this time *Ptc1* and *Gli1* are localized to the mesenchyme directly around the emerging buds [4]. Several laboratories have studied the role of *Hh* signaling in prostate development. The earliest studies used a polyclonal antibody to neutralize *Shh* signaling and noted inhibition of prostate morphogenesis in the grafted UGS [3]. Subsequent studies showed prostate development was not dependent on *Shh* signaling since the UGS from a *Shh* null embryo exhibited normal prostate morphogenesis when grafted [5,6,7]. However; we then observed that *Ihh* expression was increased in the *Shh* null UGS and provided functional redundancy that rescued *Hh* signaling [12]. Whether *Hh* signaling is strictly required for prostate morphogenesis remains to be determined. 

## 4. Effects on Growth and Ductal Morphogenesis

Initially it was reported that chemical inhibition of *Hh* signaling in the cultured UGS resulted in decreased epithelial proliferation and branching [4]. However, later studies reported a myriad of effects on branching during experimental approaches to increase or decrease *Hh* signaling in the cultured postnatal prostate[5,6,7]. Wang reported inhibition of *Hh* signaling increased overall epithelial proliferation [5]. However, the effects were regionalized along the duct. In fact, epithelial proliferation was significantly and selectively decreased in the distal, less differentiated part of the duct [5,6]. We postulated that inconstant findings reported by different laboratories might be attributed to the studies being done at different stages of prostate development [5]. This was subsequently shown to be the case. Using a combination of chemical inhibition of *Hh* signaling and transgenic activation of *Hh* signaling, it was shown that paracrine *Hh* signaling promotes epithelial proliferation and budding prenatally while inhibiting epithelial proliferation and branching postnatally. These differential effects were mirrored by stage-specific differences in *Hh* target gene regulation [8]. These studies established paracrine Hh signaling as a primary regulator of ductal morphogenesis and epithelial proliferation. Stage dependent effects are associated with an evolving palette of target gene regulation and reveal that the effects of paracrine Hh signaling are dependent upon the mesenchymal microenvironment. 

Beachy and colleagues performed elegant studies localizing Hh ligand expression and pathway activity during branching morphogenesis in the regenerating prostate [13]. They found both Shh and Ihh to be expressed at significant levels but observed focally decreased Hh expression and pathway activity at sites of ductal branching. Manipulations to decrease Hh pathway activity increased ductal branching and this was associated with increased stromal expression of hepatocyte growth factor (HFG) a positive regulator of ductal branching. Ongoing studies are yielding additional evidence of cross-talk between Hh and other regulators of prostate development. Shh expression is diminished in Sox9 genetically deficient mice [14] but prolonged in FoxA1 deficient mice [10]. Pu et al. [11] demonstrated that exogenous Shh decreased FGF10 expression in cultured prostate tissues.

## 5. Autocrine Signaling

**Figure 2:**
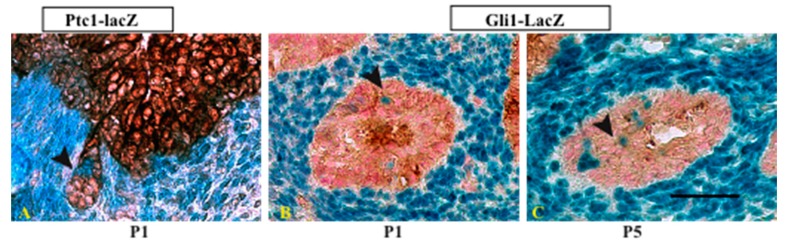
X-gal/PanCK staining of P1/P5 prostate from *Gli1^lacZ^* and *Ptc1^lacZ^* mice showed strong positive beta-gal activity (blue) in the mesenchyme generally and in a limited number of epithelial cells (brown) in the nascent ducts (arrowheads). N = 3, scale bar = 40 µm.

We performed preliminary studies to examine the role of autocrine Hh signaling in the prostate. Using *Ptc1^lacZ^* and *Gli1^lacZ^* reporter mice to localize Hh pathway activation, we observed robust expression of *Ptc1^lacZ^* and *Gli1^lacZ^* in the urogenital mesenchyme. Most epithelium was unstained, but we observed strong lacZ staining in a small number of epithelial cells in the nascent prostate ducts at P1 and P5 (Figure 2). Staining of the adult prostate showed relatively infrequent and scattered epithelial staining (data not shown). These observations suggest that autocrine Hh pathway activation occurs in a small number of epithelial cells specifically during prostate development.

**Figure 3:**
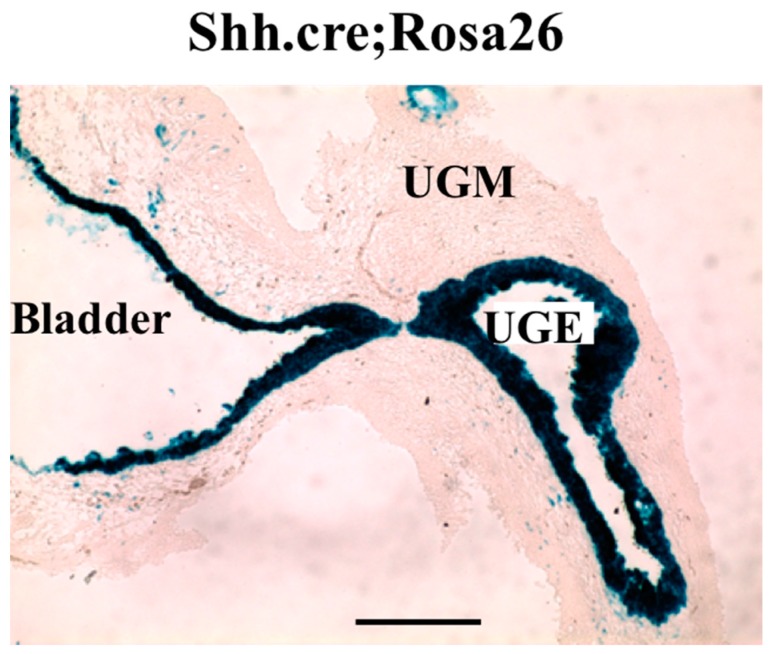
Staining for beta-gal in E16 UGS of *Shh^cre^*;Rosa26R mice. Note efficient recombination through the UGS epithelium (UGE) and bladder epithelium. UGM: UGS mesenchyme; n = 3.

**Figure 4:**
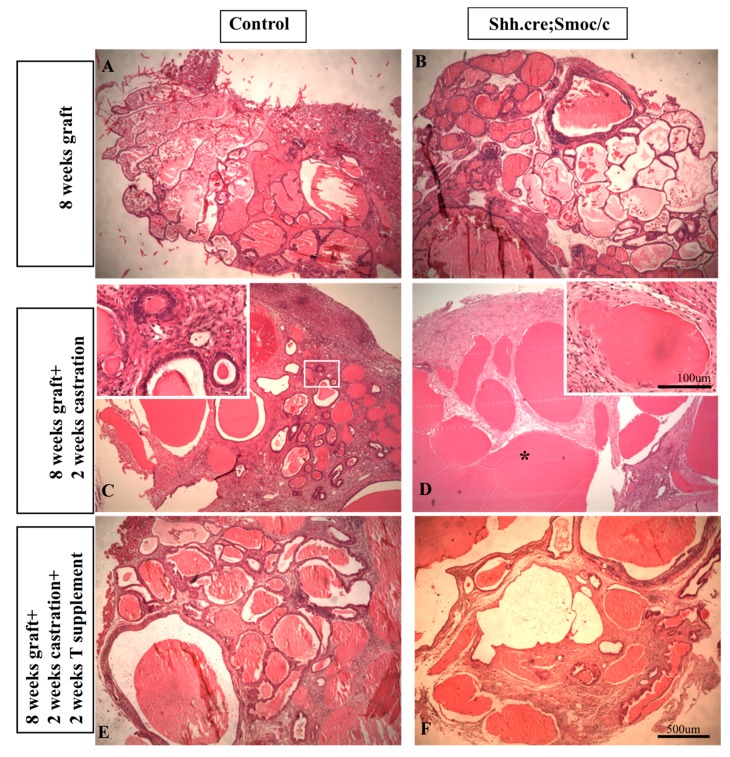
In vivo subcapsular grafts of P1 UGS from control and *Shh^cre^*;*Smo^c/c^* mutants. Histologic appearance after eight weeks growth (top), two weeks after castration (middle) and two weeks after testosterone supplement. n = 3.

To examine the role of autocrine signaling specifically, we abrogated epithelial Hh signaling by conditional deletion of the essential pathway component Smoothened (*Smo*) using a *Shh^cre^* construct B6.Cg-Shh^tm1(EGFP/cre)Cjt^/J (stock 005622). We have previously shown that *Shh* mRNA expression in the developing prostate is restricted to the epithelium [3,4]. When the *Shh^cre^* mouse was bred to the *lacZ* reporter mouse Rosa26 (B6.129S4-Gt(ROSA)26Sor<tm1Sor>/J 003474) X-gal staining of the UGS from *Shh^cre^*;Rosa26 mice exhibited robust positive staining restricted to the epithelium (Figure 3) and Mehta et al. [15]. *Shh^cre^* was bred to Smo^c/c^ (Smo^c/c^: STOCK Smo^tm2Amc/J^ 004526) to selectively delete *Smo* from all cells expressing *Shh*, including the UGS epithelium. *Shh^cre^*;Smo^c/c^ mutant pups were small with abnormalities in limb, digits and tail and died as newborns (data not shown). The P1 UGS from *Shh^cre^*;Smo^c/c^ mutants and controls were grafted under the renal capsule of adult nude males [12,16]. Histologic examination after eight weeks showed well-developed ductal structures and differentiated epithelium and stroma in both groups (Figure 4, top panels). 

## 6. Prostate Regeneration

Development and maintenance of the prostate is exquisitely dependent on androgen. Castration of the adult results in widespread epithelial apoptosis and glandular involution. Regeneration of the prostate occurs with administration of exogenous testosterone and involves robust cell proliferation, branching morphogenesis and complete reconstitution prostate architecture and differentiation. The prostate’s unique regenerative capacity has stimulated interest in the role of progenitor cells in this process and the study of prostate stem cells has been an active area of investigation. We performed studies showing that castration induces a selective expansion of slow-cycling progenitor cells. The implication of this finding is that these are “androgen-independent” progenitor cells that could play an important role in prostate regeneration. Interestingly, we found that both Shh and Gli expression are increased significantly in response to castration [17] and observed robust Hh signaling when progenitor cells are grown in anchorage independent culture [18]. These findings suggested a role for Hh signaling in progenitor cell expansion and the capacity for prostate regeneration.

**Figure 5:**
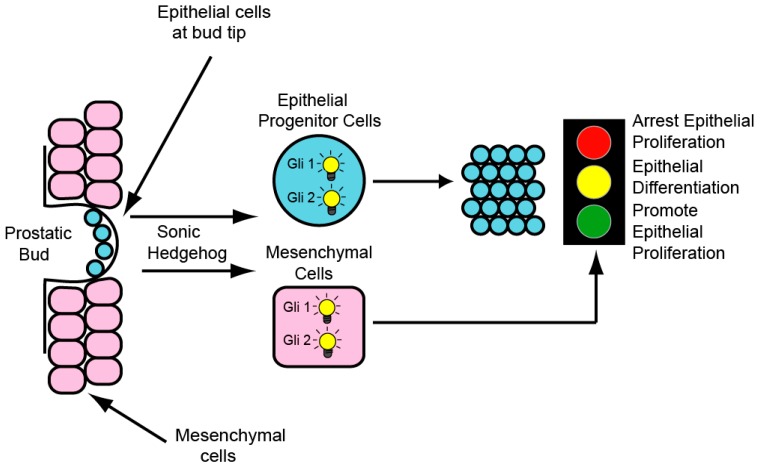
Hh signaling in the developing prostate involves both autocrine and paracrine signaling. Autocrine signaling drives proliferation of (androgen independent) progenitor cells at the bud tip. Paracrine signaling elicits a complex transcriptional response, determined by the stage of development, that exerts a variety of effects on epithelial proliferation and differentiation. In the prenatal prostate, Hh pathway activity drives epithelial proliferation and ductal growth. In the postnatal prostate, Hh pathway activity inhibits epithelial proliferation and ductal growth. Androgen independent progenitor cells generated by autocrine Hh signaling during development are able to survive castration and enable prostate regeneration in response to testosterone supplement.

A role for Hh signaling in prostate regeneration has been previously recognized [19] demonstrated that inhibition of Hh signaling abrogated prostate regeneration but did not distinguish between autocrine and paracrine signaling. Using grafted P1 UGS from *Shh^cre^*;Smo^c/c^ mutants and controls, we characterized the effect of disrupted autocrine signaling on the response to castration and testosterone supplement. Eight weeks after grafting, mice were castrated and examined 14 days later. We observed a truly striking difference. Whereas the control grafts exhibited well-formed ducts with cuboidal epithelium, the mutant grafts lacked well defined cuboidal epithelial cells. Grafted tissues castrated x 14 days and then testosterone supplemented x 14 days revealed an equally exciting disparity in re-growth. Whereas control grafts recovered normal morphology comparable to the intact tissue, the mutant grafts exhibited an atrophic appearance with fewer, dilated ductal lumen and minimal epithelial re-growth (Figure 4, bottom panels). These previously unpublished observations indicate that disruption of autocrine signaling may not perturb prostate development but exaggerates the response to castration and impairs regeneration in response to testosterone. A proposed model for the complex roles of autocrine and paracrine Hh signaling in prostate development and capacity for regeneration is shown (Figure 5). 

## 7. Prostate Cancer

**Figure 6:**
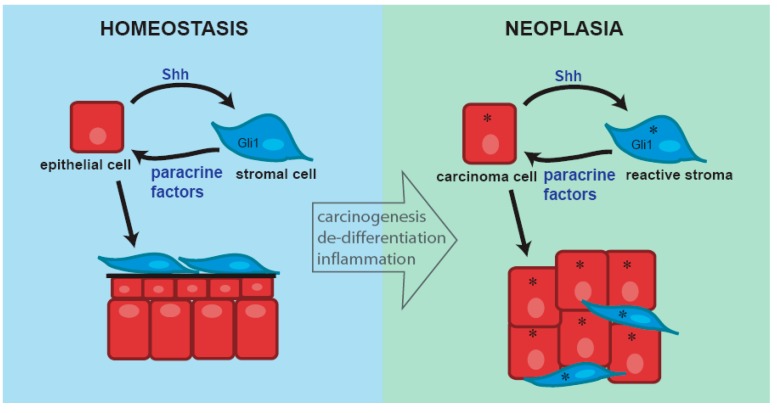
The target genes activated by paracrine Hh signaling in the adult prostate and the effect on epithelial/tumor cell proliferation is determined by the phenotype of the stroma. In the normal adult prostate and prostate cancers with a normal fibroblastic stroma the target gene profile resembles that of the postnatal prostate and the effect of paracrine Hh signaling is homeostatic. In prostate cancers with a so-called “reactive” (myofibrobastic) stroma, the target gene profile resembles that of the prenatal prostate and the effect of paracrine signaling is to promote epithelial/tumor cell proliferation. Autocrine signaling in tumor stem cells may enable these cells to survive castration and enable androgen independent tumor growth.

Hh signaling is active in human prostate cancer and evidence for both autocrine and paracrine signaling in human prostate cancer has been presented. This appears to involve both ligand-dependent signaling as well as non-canonical pathway activation. Paracrine signaling has been modeled using xenograft studies and shown to promote tumor growth [20]. Interestingly, a correlation analysis of Shh target gene expression in human prostate cancer revealed dichotomous expression of prenatal and postnatal target genes in the tumors. In those tumors with a myofibroblastic stroma resembling the mesenchyme of the developing prostate, Shh and Gli1 expression correlated with expression of prenatal target genes. In those with a more mature, myofibroblast poor stroma, there was correlation with expression of postnatal target genes. These findings suggest that the effect of paracrine signaling in prostate cancer as in development, is dependent upon the stromal context and microenvironment in which it occurs. The role of autocrine signaling in prostate cancer is debated. Several studies appeared to show autocrine signaling in prostate cancer cell lines cultured in vitro [19,21] but this has been contested by two other studies [22,23]. Hh signaling appears to be consistently up-regulated in metastatic and hormone refractory prostate cancer and several studies suggest that Hh pathway activation may be a critical step in tumor metastasis and progression to androgen independence [19,21,24]. A proposed model for the roles of autocrine and paracrine Hh signaling in prostate cancer is shown (Figure 6). 

## 8. Summary and Conclusions

Shh is the dominantly expressed ligand in the developing prostate. In absence of Shh, Ihh expression is up-regulated and provides functional compensation. In the regenerating adult prostate, both Shh and Ihh contribute to pathway activity and inhibit ductal branching by restricting the expression of HGF.

Paracrine signaling in the developing prostate plays an important role in regulating growth and ductal development. The growth effects and target gene regulation are stage-dependent.

There is evidence for autocrine signaling in the developing prostate but its role is unclear. Preliminary studies suggest it may have an important role in the capacity to survive castration and enable ductal regeneration. 

The role of Hh signaling in development and regeneration is recapitulated in cancer. There is strong evidence for paracrine signaling in prostate cancer. The effect of paracrine signaling may be critically dependent on the phenotype of the tumor stroma. This has major implications for the design of clinical trials of Hh inhibitors in prostate cancer. The role of autocrine signaling remains ill-defined. Our studies suggest a link between autocrine signaling and the capacity of epithelial cells to survive castration and there is a suggestion of pathway activation in high grade, androgen independent prostate cancer. It is as yet unclear if this is ligand-dependent or due to non-canonical activation mechanisms.
